# Deep ocean water alters the cholesterol and mineral metabolism of squid *Todarodes pacificus* and suppresses its weight loss

**DOI:** 10.1038/s41598-023-34443-x

**Published:** 2023-05-10

**Authors:** Kaito Hatano, Masa-Aki Yoshida, Jun Hirayama, Yoichiro Kitani, Atsuhiko Hattori, Shouzo Ogiso, Yukina Watabe, Toshio Sekiguchi, Yoshiaki Tabuchi, Makoto Urata, Kyoko Matsumoto, Akihiro Sakatoku, Ajai K. Srivastav, Kenji Toyota, Hajime Matsubara, Nobuo Suzuki

**Affiliations:** 1 Noto Marine Laboratory, Institute of Nature and Environmental Technology, Kanazawa University, Ogi, Noto-cho, Ishikawa, 927-0553 Japan; 2 Marine Biological Science Section, Education and Research Center for Biological Resources, Faculty of Life and Environmental Science, Shimane University, Oki, Shimane 685-0024 Japan; 3 Department of Clinical Engineering, Faculty of Health Sciences and Division of Health Sciences, Graduate School of Sustainable Systems Science, Komatsu University, Komatsu, Ishikawa 923-0961 Japan; 4 Department of Biology, College of Liberal Arts and Sciences, Tokyo Medical and Dental University, Ichikawa, Chiba 272-0827 Japan; 5 Life Science Research Center, University of Toyama, Sugitani, Toyama, 930-0194 Japan; 6 Institute of Noto Satoumi Education and Studies, Ogi, Noto-cho, Ishikawa, 927-0553 Japan; 7 School of Science, Academic Assembly, University of Toyama, Gofuku, Toyama, 930-8555 Japan; 8 Department of Zoology, D.D.U. Gorakhpur University, Gorakhpur, 273-009 India; 9 Noto Center for Fisheries Science and Technology, Kanazawa University, Ossaka, Noto-cho, Ishikawa, 927-0552 Japan

**Keywords:** Biochemistry, Physiology, Zoology

## Abstract

This study is the first to demonstrate that deep ocean water (DOW) has physiological significant effects on squid. After 36 h of rearing squids, those reared with DOW had significantly higher total and free cholesterol levels and lower alanine transaminase activity in hemolymph as compared with those reared with surface sea water (SSW). SSW rearing also resulted in 6.95% weight loss, while DOW rearing caused only 2.5% weight loss, which might be due to liver metabolism suppression. Furthermore, both monovalent (sodium, chloride, and potassium ions) and divalent (calcium, inorganic phosphorus, and magnesium ions) ions in hemolymph were elevated when reared with DOW compared to those when reared with SSW. A study of genes expressed in the brain revealed that five genes were specifically remarked in DOW rearing. Most altered genes were neuropeptides, including those from *vasopressin* superfamily. These neuropeptides are involved in cholesterol and/or mineral metabolisms and physiological significant effects on squid. This study is the first report the effects of DOW on cholesterol and mineral metabolism of squid and will contribute to squid aquaculture using DOW.

## Introduction

Deep ocean water (DOW) is cold, salty water found 200 m below the Earth's ocean surface. It has three major characteristics, low temperature (around 5–9 °C), rich inorganic nutrients (nitrogen, phosphorus, and silicate), and cleanliness (minimal to no bacteria activities and less photosynthesis of plant plankton), making it applicable for various uses^1,2^. Mineral components (magnesium ion: Mg^2+^, calcium ion: Ca^2+^, chromium ion, vanadium ion etc.) in DOW were reported to have positive effects on human health^1^. For example, human subjects drank 1050 mL of DOW daily for 6 weeks, and blood tests showed a decrease in serum total cholesterol and low-density lipoprotein–cholesterol levels^3^. Additionally, the total serum cholesterol and triacylglycerol decreased in high-fat/cholesterol-fed hamsters^4^. Mg^2+^ included in DOW has an important role in lipid metabolism^1,5^. DOW supplemented with high Mg^2+^ concentrations (341.3 mg/L) reduced both serum and liver triglyceride and cholesterol levels in nonalcoholic fatty liver disease mice fed with high-fat diet^5^. Based on a mammalian investigation of DOW, DOW influences lipid metabolism and possesses healthy effects.

For aquaculture, the growth of seaweeds^6,7^ and shrimp^8^ was promoted by breeding in DOW as compared with those bred in surface sea water (SSW). The germiling growth rate of brown alga, *Sargassum fusiforme*, kept with DOW was 2.7 times higher than those kept with SSW^7^. The growth of juvenile sporophytes of *Eisenia arborea* and *Eisenia cava* reared with DOW was also faster^6^. Pelagic shrimp *Sergia lucens* that lives in deep sea can be kept for a long time when reared with DOW^8^; it could be kept for an average of only 13 days with SSW and 58.8 days with DOW. The shrimp could be kept for up to 185 days with DOW^8^.

*Todarodes pacificus* (Fig. S1), the Japanese common squid, is distributed in the surface and middle layers of nearshore waters from the Sea of Okhotsk north to the Sea of Japan and East China Sea. This squid is in the highest demand in Japan and Asian region; it is used not only fresh, but also in various processed foods, such as surume (dried squid) and shiokara (salted squid). However, the technology for rearing this squid has not been developed.

Our recent study found that DOW reduced the stress of marine teleost (Japanese flounder *Paralichthys olivaceus*) which was grown under high density condition^9^. In the study, kynurenine, a component existing in DOW, was identified as the responsible factor for the stress-reducing effect of DOW^9^. These findings suggest the positive effect of DOW on the physiological traits of the squid. To test this possibility, the current study compared changes in hemolymph composition and mRNA expression in the brain, as well as those in the body weight between DOW- and SSW-reared squids in the condition of identical water temperature.

## Results

### Changes in hemolymph components after rearing squids in SSW or DOW

**Figure 1:**
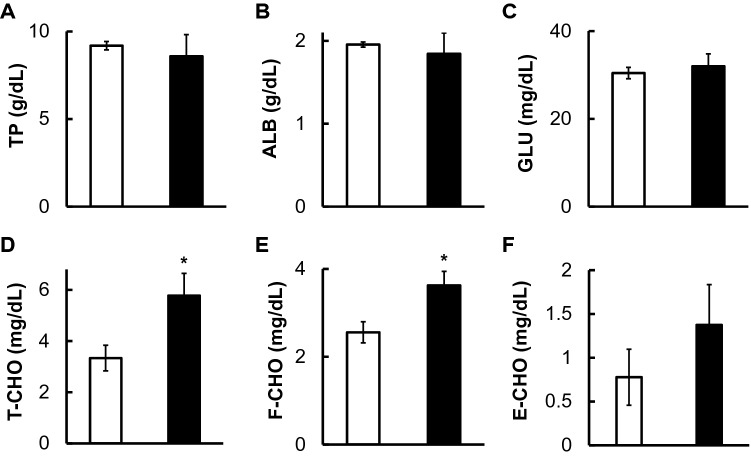
Changes in total protein (TP) (g/dL) (**A**), albumin (ALB) (g/dL) (**B**), glucose (GLU) (mg/dL) (**C**), total cholesterol (T-CHO) (mg/dL) (**D**), free cholesterol (F-CHO) (mg/dL) (**E**), and ester-type cholesterol (E-CHO) (mg/dL) (**F**) in hemolymph after rearing squids with SSW (White bar, n = 9) or DOW (Black bar, n = 10). *P < 0.05.

**Figure 2:**
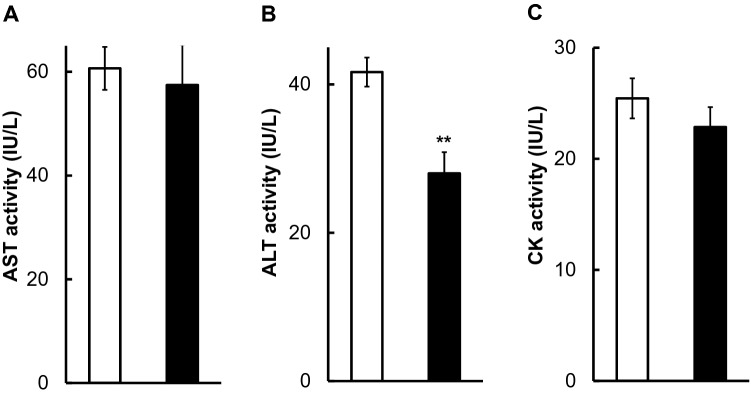
Changes in activities of aspartate transaminase (AST) (IU/L) (**A**), alanine transaminase (ALT) (IU/L) (**B**), and creatine kinase (CK) (IU/L) (**C**) in hemolymph after rearing squids with SSW (White bar, n = 9) or DOW (Black bar, n = 10). **P < 0.01.

The total protein (TP), albumin (ALB), and glucose (GLU) levels in squid hemolymph did not change between DOW and SSW rearing (Fig. 1), while cholesterol metabolism was significantly changed. The total cholesterol (T-CHO) and free cholesterol (F-CHO) levels in the hemolymph of squids reared with SSW were significantly lower than in those reared with DOW, although ester-type cholesterol (E-CHO) did not significantly change (Fig. 1). Triglycerides were not detected in the squid hemolymph at least present conditions. Those reared with DOW had significantly lower hemolymph alanine transaminase activity (ALT) as compared with those reared with SSW (Fig. 2). No significant difference was found in the hemolymph aspartate transferase (AST) and creatine kinase (CK) activities of squids kept with DOW and SSW (Fig. 2). In addition, changes in body weight before and after rearing in SSW or DOW are shown in Table S1. Interestingly, DOW rearing only caused 2.5% weight loss, while SSW rearing resulted in 6.95% weight loss.

### Changes in hemolymph mineral concentrations after rearing squids in SSW or DOW

**Figure 3:**
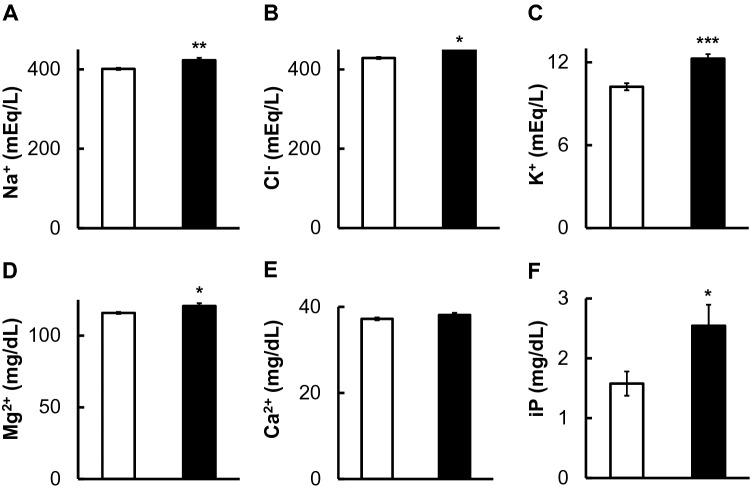
Changes in Na^+^ (mEq/L) (**A**), Cl^−^ (mEq/L) (**B**), K^+^ (mEq/L) (**C**), Mg^2+^ (mg/dL) (**D**), Ca^2+^ (mg/dL) (**E**), and iP (mg/dL) (**F**) in hemolymph after rearing squids with SSW (White bar, n = 9) or DOW (Black bar, n = 10). *P < 0.05; **P < 0.01; ***P < 0.001.

Monovalent ions (Na^+^, Cl^−^, and K^+^) and divalent ions (Mg^2+^ and Ca^2+^) in SSW and DOW showed almost the same values (Table S2). However, Na^+^, Cl^−^, and K^+^ levels in the hemolymph of squids reared with DOW were significantly higher than in those reared with SSW (Fig. 3A–C). The concentration of hemolymph Mg^2+^ in squid reared with DOW was significantly higher than in those reared with SSW (Fig. 3D). In the case of Ca^2+^, the hemolymph Ca^2+^ level of squids reared in DOW tended to be higher than those of squids reared in SSW (Fig. 3E). The hemolymph inorganic phosphorus ion (iP) level of squid kept with DOW was significantly higher than in those reared with SSW, as Mg^2+^ did (Fig. 3F).

### Changes in mRNA expression in the brain after rearing squids in SSW or DOW

**Figure 4:**
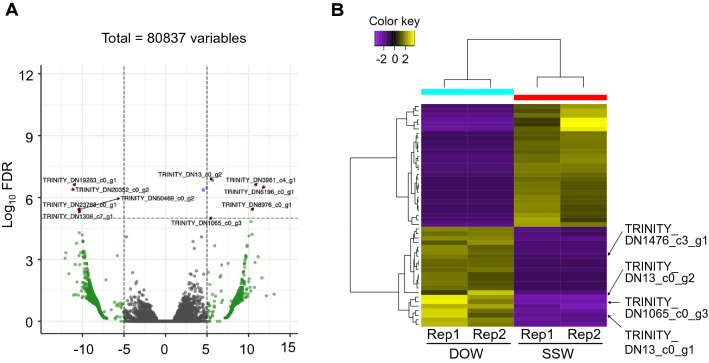
Changes in gene expression in the squid brain after rearing squids with SSW or DOW. (**A**) Volcano plot in the brains of squid after rearing with SSW or DOW. In the brains of squids reared with DOW, transcript IDs with log_2_ fold change > 5.0 and false discovery rate (FDR) > 10^−6^ are shown. (**B**) Heatmap and hierarchical clustering by differentially expressed genes (P < 0.001) between SSW and DOW conditions.

Variations in expression (volcano plot) in the brains of squid reared with SSW and DOW are shown in Fig. 4A. Transcript IDs with significant changes between SSW and DDW (LogFC > 5.0 and false discovery rate [FDR] > 10^−6^) are shown in Fig. 4A. Squid reared in DOW had changes in genes expressed in the brain.

**Figure 5:**
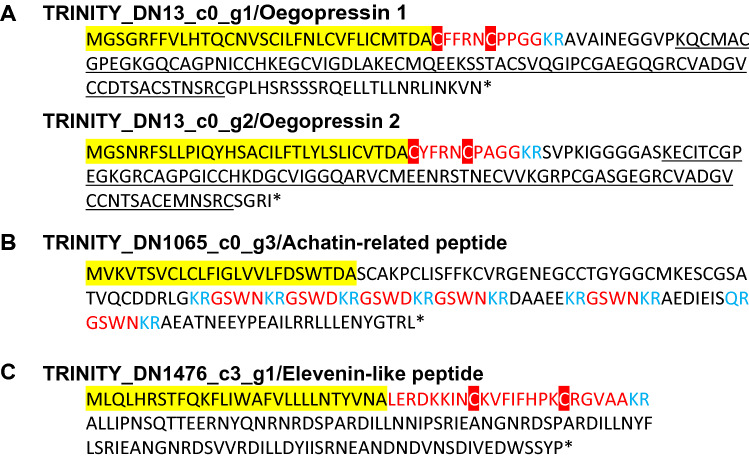
Oegopressins (**A**), Achatin-related neuropeptide (**B**), and Elevenin-like peptide (**C**) of the Japanese common squid. (**A**) Predicted amino acid sequences of *oegopressin 1* and *2*. Red font, putative mature peptide; blue font, putative peptidase cleavage sites; yellow highlight, putative signal peptide; red highlight, conserved cysteine residues for S–S bond formation; each underline shows the sequence of neurophysin present following the mature peptide. (**B**) Predicted amino acid sequences of *Todarode* achatin-related. Red font, putative mature peptide; blue font, putative peptidase cleavage sites; yellow highlight, putative signal peptide. (**C**) Predicted amino acid sequences of *Todarode* elevenin-like. Red font, putative mature peptide; blue font, putative peptidase cleavage sites; yellow highlight, putative signal peptide; red highlight, conserved cysteine residues for S–S bond formation.

Figure 4B shows a heat map with hierarchical clustering obtained by Trinity utility. Based on hierarchical clustering analysis, we found 50 genes whose expression varied significantly by DOW and SSW rearing (Table S3). Among these genes, there were five protein-coding genes whose amino acid-coding regions could be inferred. One was an unknown gene with unknown function, while the other four were neuropeptides (Oegopressin 1 and 2: Fig. 5A; Achatin-related peptide: Fig. 5B; Elevenin-like peptide; Fig. 5C). All these neuropeptide genes were upregulated when reared with DOW (Fig. 4B).

## Discussion

This study is the first to demonstrate that DOW has physiological significant effects on Japanese common squid *T. pacificus*. After 36 h of rearing squids, those reared with DOW had significantly higher T-CHO and F-CHO levels and lower ALT activity in hemolymph as compared with those reared with SSW (Fig. 1). The ALT activity, a liver marker^10–12^, also decreased in DOW rearing (Fig. 2), suggesting that liver metabolism was reduced and hemolymph cholesterol levels remained high. Additionally, their pre- and post-experimental weights were measured (Table S1). The average weight of nine squids reared with SSW decreased from 148.2 to 137.9 g, while the average weight of those reared with DOW changed from 148 to 144.3 g, indicating a small percentage reduction (− 2.5%) in weight. Those reared with DOW had a reduced weight loss by suppressing liver metabolism. On the other hand, their hemolymph AST and CK levels, which are markers of cardiac and skeletal muscle^11,13–15^, did not significantly decrease, possibly because they were constantly moving their muscles to swim.

In this study, DOW rearing affected mineral metabolism in squid. Both monovalent (Na^+^, Cl^−^, and K^+^) and divalent ions (Mg^2+^ and Ca^2+^) in hemolymph were elevated when reared with DOW compared to those reared with SSW (Fig. 3). Mineral ions other than Ca^2+^ were significantly elevated after DOW rearing (Fig. 3). Since Ca^2+^ plays an important role in squid neural activity^16,17^, this ion may be regulated by a different mechanism.

A study of genes expressed in the brain revealed that five genes were specifically remarked in DOW rearing (Figs. 4 and 5). Most altered genes were neuropeptides, including *oegopressins* superfamily, *achatin-related peptide*, and *elevenin-like peptide*, implying that they significant physiological effects on squid.

**Figure 6:**
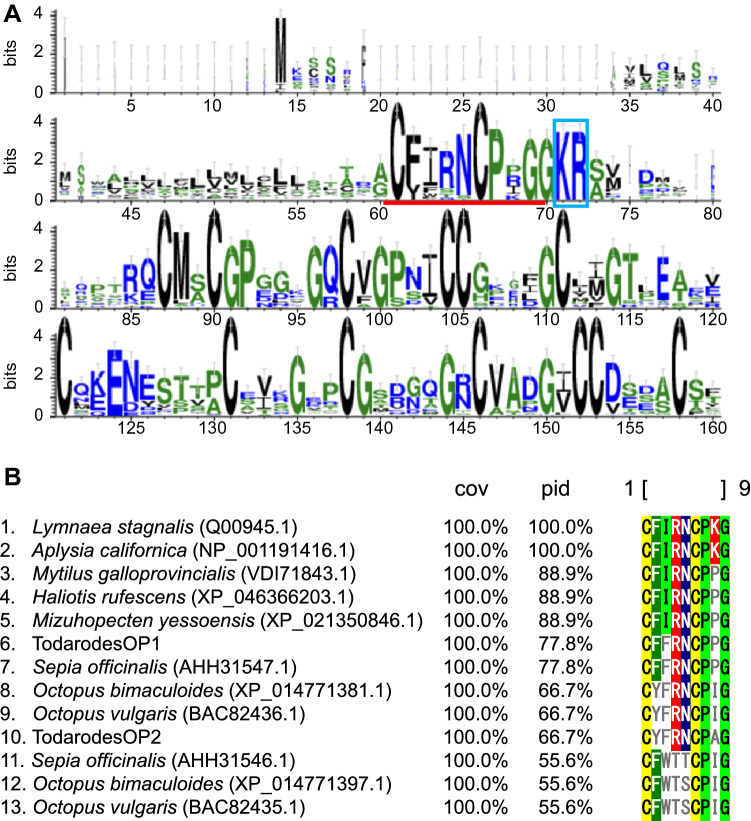
Logo representation (**A**) and sequence alignment of mature peptides (**B**) Vasopressin/Oxytocin superfamily neuropeptides. (**A**) Logo representation of Vasopressin/Oxytocin superfamily neuropeptides based on a sequence alignment of top 50 homologs by webBLASTP to Oegopressin 1 and 2. Red underline, putative mature peptide; blue rectangle, putative peptidase cleavage sites. (**B**) Sequence alignment of mature peptides of selected Vasopressin/Oxytocin homologs from mollusks.

In the Octopus species, two peptides of Octopressin and Cephalotocin including Vasopressin/Oxytocin superfamily have been isolated and identified from the rectum and nervus tissues in *Octopus vulgaris*, respectively^18,19^. Our determined two peptides belonged to Vasopressin/Oxytocin superfamily (Fig. S2 and Table S4). Sequence alignment by MAFFT showed that our determined peptides were composed of nine amino acid residues containing consensus cysteine residues as well as other bilaterian Vasopressin/Oxytocin peptides (Fig. 6A). Since these types of peptides are the first to be discovered in open-eyed squids (Oegopsids), we name it Oegopressin. The present study is the first report showing the expression of *oegopressins* in squid. The *octopressin* and *cephalotocin* genes, like the Vasopressin/Oxytocin family, were known to have evolved through duplication^20^. Both peptides in this study showed a similar degree of homology compared to the previously known Conopressin (*Lymnaea stagnali*: No. 1, Fig. 6B). The three previously known Cephalotocins (Nos. 11, 12, and 13, Fig. 6B) have a second phenylalanine and a third tryptophan, but none of the peptides found in this study are identical to these. Therefore, we concluded both novel *Todarodes* peptides are Octopressin homologs and determined Oegopressin 1: CFFRNCPPG (No. 6, Fig. 6B) and Oegopressin 2: CYFRNCPAG (No. 10, Fig. 6B) in squid. Whether other squid species besides the common squid have a separate Cephalotocin homolog will require further investigation of the genome sequence in more species in the future.

Both coding sequences are characterized by the presence of an additional functional Neurophysin sequence behind the mature peptide (each underline in Fig. 5A). In octopus, *Octopus vulgaris*, both *octopressin* and *cephalotocin* mRNA were expressed in the esophageal brain^19^. This fact is agreement with our RNA-sequencing results. After 1 day administration Octopression into octopus, the hemolymph osmolality and Ca^2+^ concentrations decreased^21^. As described above, the fact that only the Ca^2+^ in hemolymph, unlike the other ions, was not significantly elevated when reared with DOW may have something to do with the action of Octopression.

**Figure 7:**
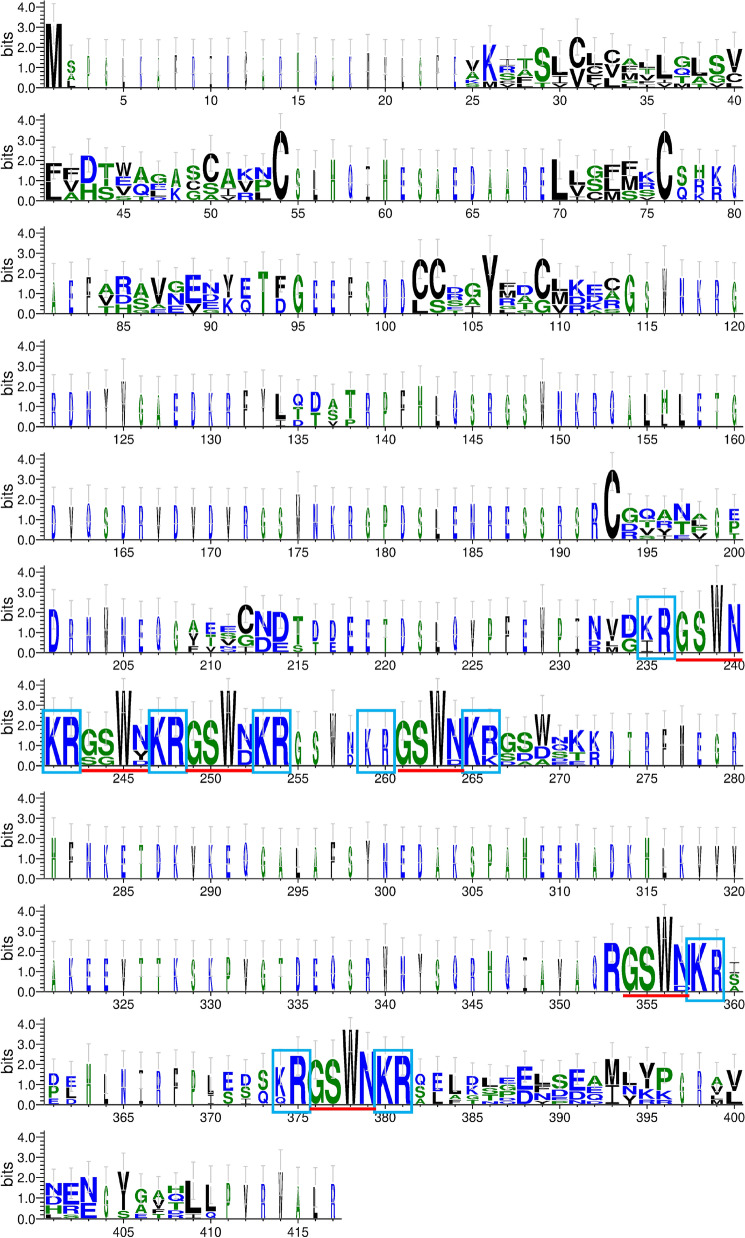
Logo representation of Achatin-related neuropeptide. Logo representation of Achatin-related neuropeptides based on a sequence alignment of webBLASTP homologs to *Todarode* achatin-related. Red underline, putative mature peptide; blue rectangle, putative peptidase cleavage sites.

Achatin-I, a tetrapeptide (Gly-d-Phe-Ala-Asp), was purified and determined from the suboesophageal and cerebral ganglia of the African giant snail, *Achatina fulica* Férussac^22^. This peptide had a bioactivity and evoked a potent neuroexcitatory effect, although Gly-l-Phe-l-Ala-l-Asp, termed Achatin-II, was ineffective on the neurons of African giant snail^22,23^. The mRNA expression of this peptide increased in the squid brain when reared with DOW. This is the first report of this peptide in a cephalopod. According to a BLAST search, only eight sequences were deposited; all had amino acid sequences encoding multiple peptides, and the sequences of mature peptides were polymorphic with GSWN or GSWD, which is also the case for squid (Figs. 5B and 7). The one coding sequence encoded six mature peptides, whereas the others encoded four to five peptides, and the peptidase excision sites were also conserved (Fig. 7). We intend to investigate the presence of D-type amino acid residues in this peptide and its bioactivity in detail.

**Figure 8:**
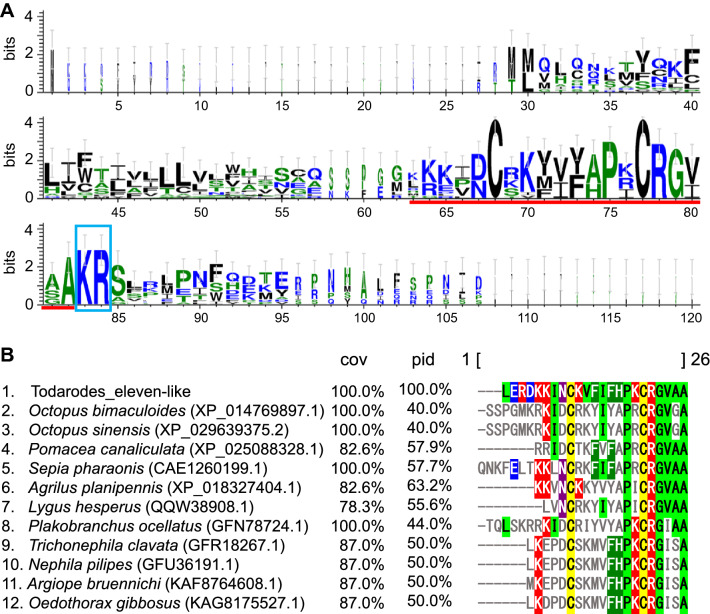
Logo representation (**A**) and sequence alignment (**B**) of Elevenin-like neuropeptides. (**A**) Logo representation of Elevenin-like neuropeptides based on a sequence alignment of webBLASTP homologs to *Todarode* Elevenin-like. Red underline, putative mature peptide; blue rectangle, putative peptidase cleavage sites. (**B**) Sequence alignment of Elevenin-like mature peptides of invertebrates.

*Elevenin* was identified as a cDNA sequence encoding a neuropeptide precursor from the L11 neuron in the abdominal ganglia of California sea hare *Aplysia californica*^24^. Thereafter, the knockdown of *Elevenin* by RNA interference caused severe cuticle melanization in the brown planthopper *Nilaparvata lugens*^25^. Furthermore, the administration of synthetic Elevenin peptide rescued the body color phenotype in *Elevenin*-dsRNAi-treated individuals and suppressed the melanization of black insects grown under natural conditions^25^. An Elevenin-like peptide (CKVFIFHPKCRGVAA) found in the squid brain may be involved in melanin metabolism in squid. This peptide codes a single mature peptide like Oegopressin 1 and 2 (Fig. 5). According to a BLAST search, 12 sequences were deposited. There was a variation in the sequence length of the mature peptide, but the consensus cysteine residues were well-conserved (Fig. 8A,B).

It is known that the Vasopressin/Oxytocin superfamily regulates mineral metabolism^26–28^. Several peptides in invertebrates are also involved in the regulation of lipid metabolism^29,30^. Thus, these peptides upregulated in the squid brain after rearing with DOW are likely to have a physiological activity in squid and regulate both mineral and lipid metabolism. In mammals, Mg^2+^ in DOW has an important role in lipid metabolism^1,5^. In mammals, brain neuropeptides may also be involved in lipid metabolism regulation by DOW. The analysis of the actions of these peptides in squid may also contribute to the effects of DOW on lipid metabolism in mammals. Thus, we would like to investigate the effects of these peptides on squid to determine their physiological effects in squid and contribute to squid aquaculture.

One important issue we raised was the mechanism underlying DOW influences the physiological traits of the squid. We have found that DOW reduced stress in marine teleosts which were grown under high density condition^9^. In addition, we have identified kynurenine, a component existing in DOW, as the responsible factor for the stress-reducing effects of DOW. Based on the findings, we expect that unknown component(s) existing in DOW would be responsible for physiological trait changes of the squid induced by DOW.

## Conclusion

DOW has significant physiological effects on *T. pacificus*. Those reared with DOW had a reduced weight loss as compared with those reared with SSW. Thus, the achievement of our research using DOW could be applied to squid rearing techniques.

## Materials and methods

### Statement on the ethical treatment of animals

This study has been conducted in compliance with recommendations of the ARRIVE Guideline^31^ for reporting in vivo experiments with research animals. All experimental protocols in this study were approved by the Animal Welfare Committee of Kanazawa University. All experiments were performed in a manner that minimized pain and discomfort.

### Animals

Japanese common squid *T. pacificus* (n = 19, 148.1 ± 5.4 g) were collected in Toyama Bay by a fisherman. To confirm the squid species, the COI gene (TRINITY_DN15407_c0_g2_i1) was cloned from the collected squid. Sequence of the cloned gene was then determined to conduct a BLAST search. As the result, the determined sequence was found to be identical to the sequence of *T. pacificus* COI gene (Fig. S3). After acclimation kept in SSW for a day at 15–16 °C for 6 h, these squids were used in the present experiments.

### Rearing squid with DOW or SSW

The squids were divided into two groups (SSW: n = 9; DOW: n = 10) and kept with SSW or DOW for 36 h at 15–16 °C. These squids were not fed bait. After rearing with SSW or DOW for 36 h, these were anesthetized with cold seawater, and hemolymph was taken from their branchial heart using a syringe. The collected hemolymph was put into a 1.5-mL tube. Then, the tube was centrifuged at 5200×*g* for 5 min. The separated hemolymph was immediately frozen and kept at − 80 °C until further use. After hemolymph sampling, each squid was dissected. The brain above the esophagus was extracted, placed in RNA*later* (Sigma-Aldrich, St. Louis, MO, USA), and stored at − 80 °C.

Additionally, changes in body weight before and after rearing were examined. Since this species cannot be reared individually, changes in the average body weight of the SSW and DOW groups were calculated instead using their individual body weight.

### Measurement of mineral concentration and hemolymph components

Hemolymph samples were sent to a commercial vendor (Oriental Yeast Co., Ltd., Tokyo, Japan), and Na^+^, Cl^−^, and K^+^ were measured through an ion electrode method using a Hitachi 7180 automatic analyzer (Hitachi High Technologies Corporation, Tokyo, Japan). Hemolymph Mg^2+^, Ca^2+^, and iP levels (mg/dL) were determined using assay kits (Mg^2+^: Mg·N, FUJIFILM Wako Pure Chemical Corporation, Osaka, Japan; Ca^2+^: Ca II, Shino-Test Corporation, Tokyo, Japan; iP: IP-II, Kyowa Medex Co., Ltd., Tokyo, Japan). TP, ALB, GLU, T-CHO, F-CHO, E-CHO, triglyceride, AST activity, ALT activity, and CK in hemolymph were measured using several kits (FUJIFILM Wako Pure Chemical Corporation).

### Analysis of mRNA expression in the brain after rearing squids with SSW or DOW

Total RNAs were isolated using a kit (RNeasy Plant Mini Kit, Qiagen GmbH, Hidden, Germany). Genomic DNA was removed using an RNase-Free DNase Set (Qiagen). A complementary DNA library was constructed and sequenced with a 150 bp paired-end module using Illumina NovaSeq 6000 (Illumina, San Diego, CA, USA). Raw sequence reads were deposited at the DNA Data Bank of Japan (DDBJ) under the DDBJ Sequence Read Archive (DRA) accession no. DRA015361. Adaptors and low-quality reads were removed using fastp v0.23.2 (default setting^32^). Subsequently, unigenes were obtained using Trinity assembly program v2.8.5^33^. Only contigs with transcript per million greater than 1.0 were filtered with Trinity utility v2.14.0 and used for subsequent analysis. Kallisto was used for mapping analysis^34^. Statistical analysis for differentially expressed genes was performed with edgeR in the Trinity utilities. Transdecoder v5.5.0 was used to estimate gene-coding regions (https://github.com/TransDecoder/TransDecoder), and eggNOGmapper v2.1.9 was used for the functional annotation of amino acid sequence data^35,36^.

Homologous sequences of neuropeptide sequences (*oegopressin 1* and *oegopressin 2*, *achatin-related peptide*, and *elevenin-like peptide*) were estimated by NCBI webBLAST (blastp) (as of November 27. 2022). Alignments of amino acid sequences were estimated with MAFFT on EMBL-EBI^37^. Mview on EMBL-EBI was used to reformat the results of MAFFT alignment. Sequence logos were generated using Weblogo3 (https://weblogo.threeplusone.com/) to show the sequence conservation at each sequence position^38,39^.

### Statistical analysis

All results are expressed as means ± standard error. The statistical significance between the control and experimental groups was assessed using an independent sample *t* test. The selected significance level was p < 0.05.

## Supplementary Information

Supplementary Information.

Supplementary Table S3.

Supplementary Table S4.

## Data Availability

The raw sequence reads were deposited at the DNA Data Bank of Japan (DDBJ) under the DDBJ Sequence Read Archive (DRA) accession no. DRA015361 (https://ddbj.nig.ac.jp/resource/sra-submission/DRA015361).
