# Supplementary material for: Deep ocean water alters the cholesterol and mineral metabolism of squid Todarodes pacificus and suppresses its weight loss
Source: Sci Rep. 2023 May 10;13:7591. doi: 10.1038/s41598-023-34443-x (PMC10172372; doi:10.1038/s41598-023-34443-x)
Supplement: Supplementary file 1 — Supplementary Information. [file 41598_2023_34443_MOESM1_ESM.pdf]

Supplementary data for

Deep ocean water alters the cholesterol and mineral metabolism of squid *Todarodes pacificus* and suppresses its weight loss

Kaito Hatano, Masa-Aki Yoshida, Jun Hirayama, Yoichiro Kitani, Atsuhiko Hattori, Shouzo Ogiso, Yukina Watabe, Toshio Sekiguchi, Yoshiaki Tabuchi, Makoto Urata, Kyoko Matsumoto, Akihiro Sakatoku, Ajai K. Srivastav, Kenji Toyota, Hajime Matsubara, and Nobuo Suzuki

This pdf file includes:

Supplementary Figures S1 to S3
Supplementary Tables S1 and S2

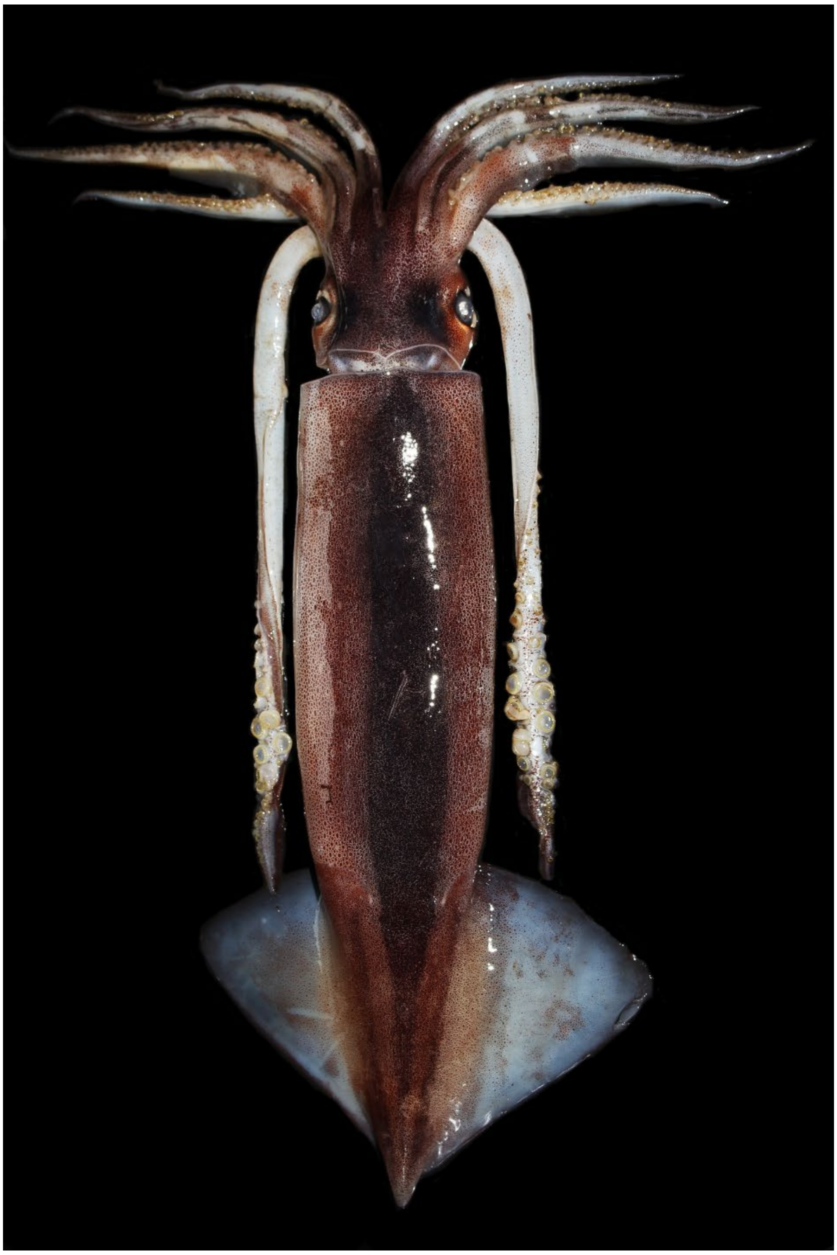

Supplementary Figure S1. Photograph of Japanese common squid *Todarodes pacificus*.

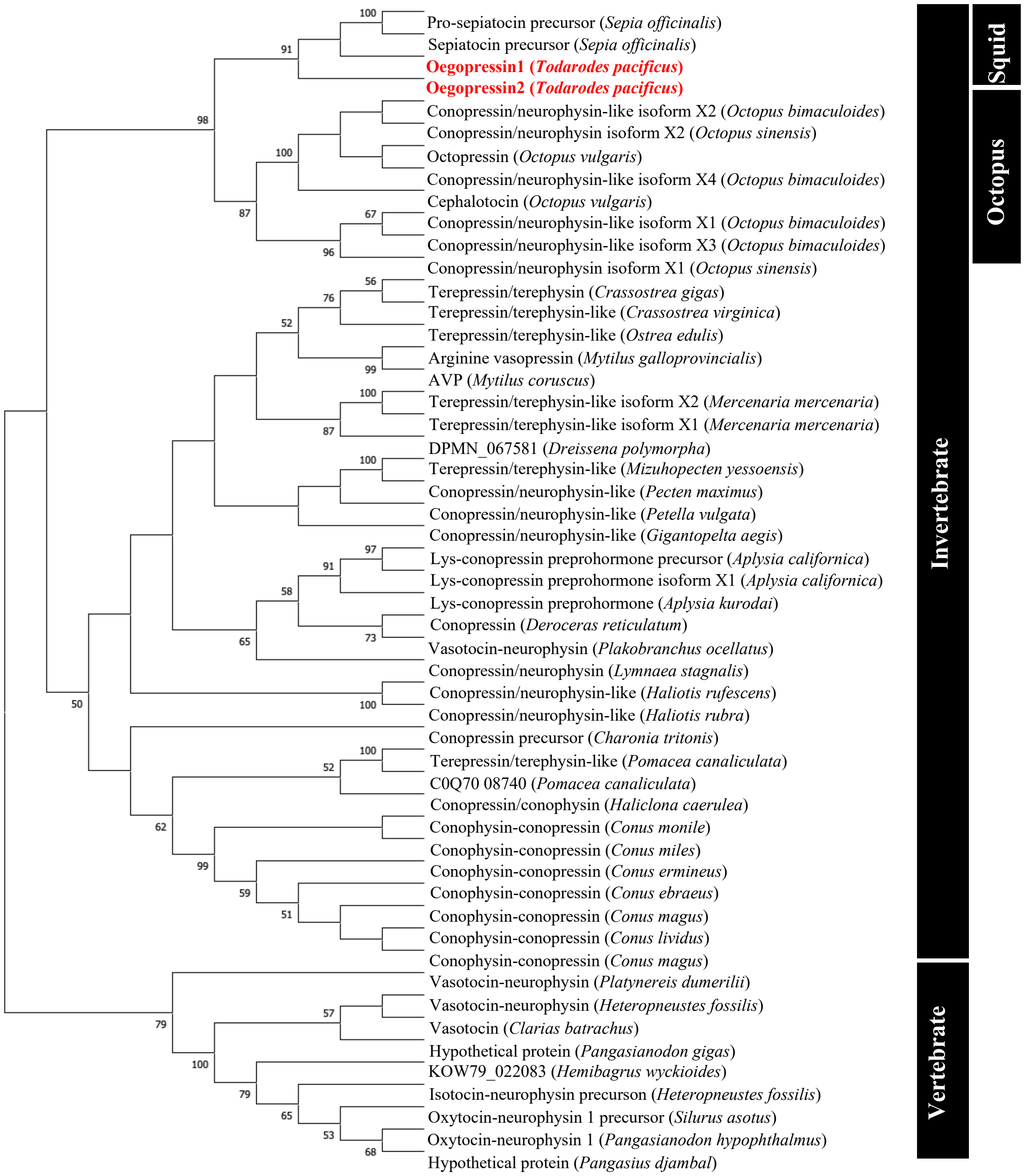

Supplementary Figure S2. Phylogeny of Vasopressin/Oxytocin superfamily in invertebrates and vertebrates. Sequences were aligned by MAFFT, and trees were generated by MEGA 11 (Tamura et al., 2021). The sequence data were constructed by maximum likelihood with bootstrapped 1000 times to estimate the confidence in the nodes. Values lower than 50 are hidden. All species names and accession numbers used in this phylogeny are listed in Table S4.

Tamura, K., Stecher, G. & Kumar, S. MEGA11: Molecular evolutionary genetics analysis version 11. *Mol Biol Evol* **38**, 3022-3027 https://doi.org/10.1093/molbev/msab120 (2021)

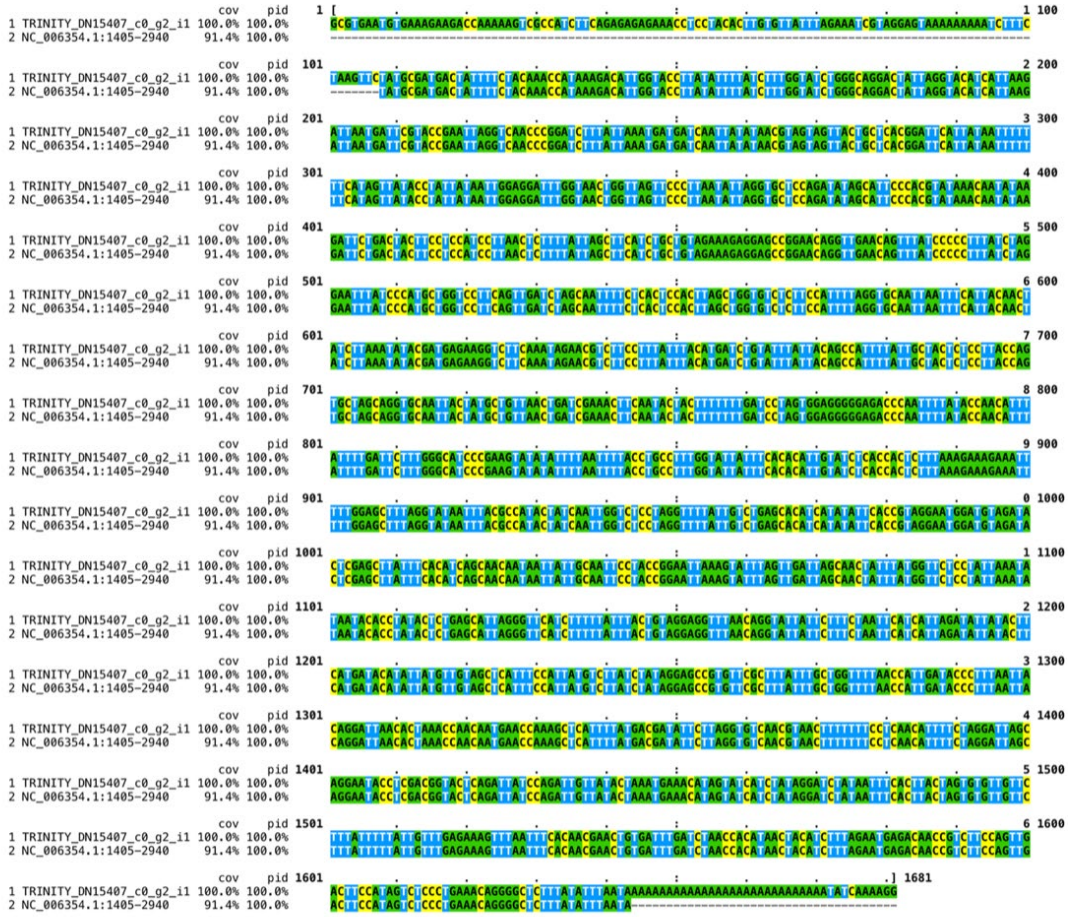

Supplementary Figure S3. Alignment analysis of mitochondrial cytochrome c oxidase subunit 1 (COI) genes used squid. The sequence of the squid COI gene (TRINITY_DN15407_c0_g2_i1) we used in the present study matched 100% with that of the squid COI gene in the Japanese common squid *Todarodes pacificus*.

Supplementary Table S1. Changes in body weight before and after rearing squids with SSW or DOW.

	**SSW**	**DOW**
**Initial (mean)**	**148.2 g**	**148 g**
**Final (mean)**	**137.9 g**	**144.3 g**
**Percentage of weight loss (%)**	**-6.95%**	**-2.50%**

Supplementary Table S2. Mineral concentrations (Na^+, Cl^-, K^+, Mg^{2+}, and Ca^{2+}) of squid reared with SSW or DOW.

	Na^+ (mEq/L)	K^+ (mEq/L)	Cl^- (mEq/L)	Mg^{2+} (mg/dL)	Ca^{2+} (mg/dL)
**SSW**	**470**	**9.9**	**470**	**129**	**41**
**DOW**	**470**	**10**	**470**	**130**	**41**
